# Research Trends and Gaps in Human Papillomavirus Vaccination Intention in South Korea: A Scoping Review

**DOI:** 10.3390/healthcare14030355

**Published:** 2026-01-30

**Authors:** Jiyeon Bark, Haejin Kim, Soyoung Seo

**Affiliations:** 1 Department of Nursing, Cheju Halla University, 38 Halladaehak-ro, Jeju-si 63092, Jeju-do, Republic of Korea; rosajy0822@naver.com; 2 Department of Nursing, Changwon National University, 20 Changwondaehak-ro, Uichang-gu, Changwon-si 51140, Gyeongsangnam-do, Republic of Korea; 3 Department of Obstetrics and Gynecology, Good Gang-An Hospital, 204-27, Bujeon-ro, Busanjin-gu, Busan 47280, Republic of Korea; ssy820920@naver.com

**Keywords:** human papillomavirus, vaccination policy, public health strategy, vaccine hesitancy, scoping review

## Abstract

**Background/Objectives:** Human papillomavirus (HPV) is a major cause of cervical, penile, anal, and oropharyngeal cancers. HPV vaccination is the most effective public health strategy for its prevention. Understanding the factors influencing vaccination intentions is critical for developing effective public health policies and improving population-level vaccine uptake. Therefore, in this scoping review, we aimed to examine HPV vaccination research conducted in Korea, identify common trends and gaps in study populations and influencing factors, and provide evidence-based recommendations for public health policies. **Methods:** We systematically searched four Korean databases—Research Information Sharing Service (RISS), DBpia, Korean Studies Information Service System (KISS), and National Digital Science Library (NDSL)—for studies published from their respective inception dates to January 2025, using “human papillomavirus,” “HPV,” “vaccination,” and “intention” as keywords. Thirty-six studies were ultimately included. Study characteristics, populations, theoretical frameworks, and key variables were extracted and analyzed using descriptive statistics and content analysis. **Results:** Of the included studies, 61.1% and 38.9% targeted vaccination-eligible individuals (adolescents and adults) and parents/guardians, respectively, with 50% focusing exclusively on women. The major factors influencing HPV vaccination intention were attitude (47.2%), subjective norms (38.9%), and perceived behavioral control (30.9%). Attitude and knowledge were critical for vaccination-eligible individuals (Direct group), whereas subjective norms were key for parents/guardians (Indirect group). **Conclusions:** Korean HPV vaccination intention research has predominantly focused on women and parents, with insufficient attention to adolescents and men. Public health strategies must employ multilevel interventions tailored to each group’s decision-making structures, including school-based programs for adolescents, gender-inclusive policies for men, and community-based approaches to address social norms among parents. These findings provide evidence for policy development aligned with the WHO cervical cancer elimination goals.

## 1. Introduction

The human papillomavirus (HPV) is primarily transmitted through sexual contact and is a leading cause of cervical, penile, anal, oropharyngeal, and genital warts [1]. HPV infection is responsible for 99.7% of cervical cancer cases, the fourth most common cancer among women globally [2]; moreover, it is estimated to account for approximately 5% of all cancers worldwide [3]. HPV vaccination is widely recognized as the most effective public health strategy for preventing HPV infections and associated diseases; notably, the World Health Organization (WHO) strongly recommends incorporating it into national immunization programs (NIPs) [4].

In South Korea, the NIP has provided free HPV vaccinations to girls aged 12–17 years since 2016. Although the initial coverage rate was approximately 61.5%, more than 80% of 12-year-old girls were vaccinated by 2024 [5]. Nonetheless, this rate remains below the WHO’s global target of “90% of girls fully vaccinated with the HPV vaccine by the age of 15” by 2025, and the vaccination rate among adolescent boys remains as low as 0.2% [6]. Considering the growing HPV-related cancer incidence in males [7,8], expanding HPV vaccination coverage for both males and females is essential for achieving herd immunity and effectively curbing HPV transmission at the population level.

Vaccination intention is a critical antecedent of vaccination behavior, reflecting an individual’s psychological readiness to receive the vaccine [9]. Previously, higher intention to vaccinate was found to be associated with increased uptake; moreover, interventions aimed at enhancing this intention directly improved coverage rates [10,11]. Consequently, identifying the factors that influence vaccination intention is an essential public health strategy to increase vaccine uptake.

HPV-related perceptions, knowledge of HPV and the HPV vaccine, health beliefs, self-efficacy, and attitudes are all associated with vaccination intention [12,13,14,15,16]. Several investigations relied on specific theoretical frameworks, such as the Theory of Planned Behavior (TPB) and the Health Belief Model (HBM), to explain vaccination intention. The selected theory often guides the focal variables and interpretive approach. TPB conceptualizes behavioral intention through three constructs—attitude, subjective norm, and perceived behavioral control—and provides a framework for understanding psychosocial determinants of preventive health behaviors, including HPV vaccination [13,17,18]. Contrastingly, HBM explains intention based on perceived susceptibility, severity, benefits, and barriers and has been widely used to examine facilitators and barriers to preventive behaviors, including HPV vaccination [14,19,20].

However, existing studies exhibit considerable heterogeneity in measurement tools, theoretical frameworks, key variables, and sample characteristics, challenging the synthesis and comparison of findings across studies. Because the researchers of these Korean studies have relied on different theoretical frameworks, particularly TPB and HBM, their findings are difficult to compare directly. Therefore, briefly outlining the characteristics of these models to clarify how they shape the selection and interpretation of key variables is essential. Furthermore, overreliance on a single theoretical model may overlook important contextual factors such as sociocultural dynamics, sex- and age-specific considerations, parental involvement in health decisions, and access to healthcare services.

To address these gaps, in this scoping review, we aim to answer the following central research questions:

1. What are the general characteristics and trends of studies on HPV vaccination intention in South Korea? (Descriptive Mapping)
2. What are the key individual and social/structural determinants of HPV vaccination intention, and how do they differ across diverse population? (Analytical Comparison)
3. To what extent have researchers of existing studies utilized theoretical frameworks, and what are the limitations of these models in explaining emerging public health factors? (Thematic Synthesis)

By answering these questions, we provide a comprehensive mapping of the current research landscape while identifying key gaps in methodology and theoretical application. Considering that South Korea is currently at a policy turning point regarding gender-neutral HPV vaccination, the identified patterns in this study will serve as a valuable reference for generating hypotheses in future longitudinal and intervention-based research. Ultimately, this evidence base will guide the design of targeted health promotion strategies and inclusive public health policies.

## 2. Materials and Methods

### 2.1. Study Design

In this scoping review, we aimed to examine Korean studies on HPV vaccination intentions, identify prevailing research trends and key determinants, and inform future directions for public health research and policy development.

This study followed the methodological framework proposed by Arksey and O’Malley [21], which consisted of five stages: (1) identifying the research question, (2) identifying relevant studies, (3) selecting studies, (4) charting the data, and (5) collating, summarizing, and reporting the results. This review included peer-reviewed studies on HPV vaccination intentions published in Korea until January 2025.

The PRISMA guidelines were followed and the review protocol was developed and registered on the Open Science Framework (OSF) and is publicly available (Registration DOI: 10.17605/OSF.IO/XBUKN).

### 2.2. Stage 1: Identifying the Research Question

The research questions in this review were developed using the PCC (Population, Concept, Context) framework. The population included all individuals studied regarding HPV vaccination intentions, including adolescents, adult men and women, parents, university students, and healthcare professionals. This concept focuses on HPV vaccination intentions and their associated determinants, including knowledge, perceptions, attitudes, self-efficacy, and health beliefs. The context encompassed both quantitative and qualitative studies as well as mixed-methods research published in Korea, including peer-reviewed journal articles and academic theses.

Based on this framework, the specific research questions addressed in this scoping review were as follows:

(1) What are the characteristics of studies on HPV vaccination intentions in Korea?
(2) What patterns can be observed when the key determinants of HPV vaccination intention are classified into individual-and social/structural-level factors?
(3) What are the differences in the determinants between the direct and indirect groups, and what are the implications of these differences for public health policy?

### 2.3. Stage 2: Identifying Relevant Studies

#### 2.3.1. Literature Search Process

This study primarily aimed to analyze research trends and key determinants of HPV vaccination intention in Korea by focusing exclusively on studies conducted in Korea. Accordingly, the literature search was restricted to Korean databases that index journal articles and academic theses published in Korea. To ensure a comprehensive collection of evidence, eligible publications were identified without any restrictions on the starting publication year, encompassing all relevant studies published from the inception of each database up to January 2025. When duplicate publications were identified, only the journal articles were included. The Korean academic databases used for the search were the Research Information Sharing Service (RISS; http://www.riss.kr), DBpia (http://www.dbpia.co.kr), Koreanstudies Information Service System (KISS), and National Digital Science Library (NDSL; https://scienceon.kisti.re.kr). The search keywords were structured into three main concepts: (1) human papillomavirus or HPV, (2) vaccination, and (3) intention. These keywords were combined using Boolean logic to ensure a comprehensive search; specifically, terms within each concept were linked with ‘OR’, and the concepts were subsequently combined using ‘AND’ (e.g., (“human papillomavirus” OR “HPV”) AND “vaccination” AND “intention”). The literature search was conducted in March 2025.

#### 2.3.2. Study Selection and Exclusion Criteria

The inclusion criteria encompassed all studies conducted in Korea that addressed HPV vaccination intention. The exclusion criteria were as follows: studies that did not address HPV vaccination intention; conference abstracts or proceedings; studies for which the full text was unavailable; studies that did not examine determinants of vaccination intention; and studies targeting Koreans residing abroad or non-Korean populations who were not beneficiaries of the NIP in Korea. To avoid redundancy, when a thesis and a journal article originated from the same research, only the journal version was included; otherwise, theses were considered eligible.

### 2.4. Stage 3: Study Selection

Overall, 279 records were identified through the database search: 82 from RISS, 70 from DBpia, 41 from KISS, and 86 from NDSL. After removal of 166 duplicate records, two researchers independently reviewed titles and abstracts from the remaining 113 studies. During screening, 77 studies were excluded because their populations, topics, or purposes did not meet the inclusion criteria; thus, 36 studies were initially selected.

**Figure 1:**
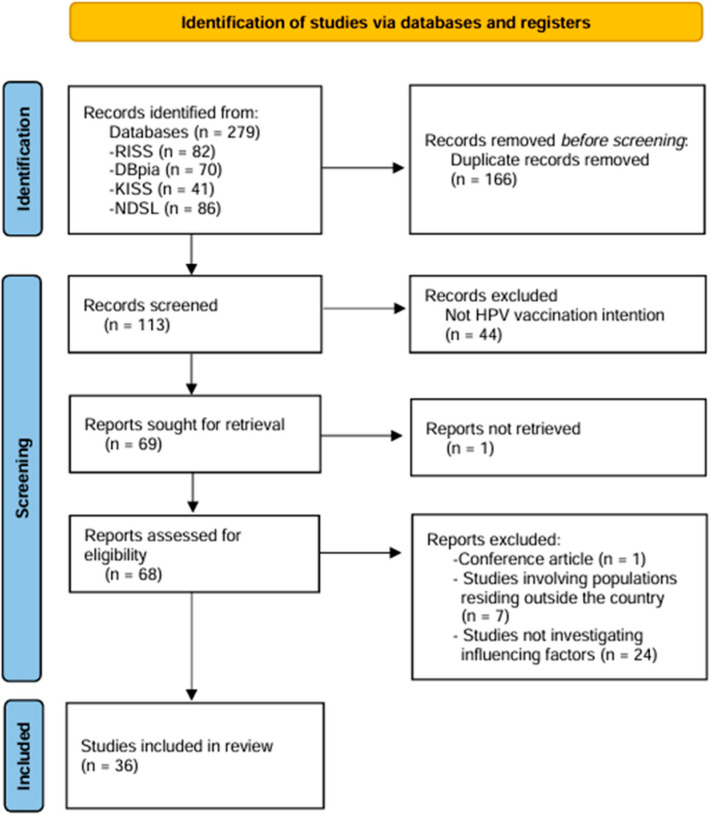
PRISMA flow diagram of study screening. RISS = Research Information Sharing Service; KISS = Koreanstudies Information Service System; NDSL = National Digital Science Library; HPV = Human papillomavirus.

For the 36 selected studies, two researchers independently conducted a methodological quality appraisal using the JBI Critical Appraisal Checklist (Supplementary Materials) for Analytical Cross-Sectional Studies [22]. All included studies demonstrated an overall acceptable level of methodological quality, and no studies were excluded solely based on the quality appraisal results. Consistent with the exploratory aim of a scoping review, the quality assessment was conducted to enhance transparency regarding the methodological characteristics of the included studies rather than to weight individual studies or to influence the synthesis. Consequently, all 36 studies were included in the final analysis (Figure 1). Of the 36 included studies, 24 were journal articles and 12 were theses.

### 2.5. Stage 4: Charting the Data

Two researchers developed an analytical framework for data extraction using Microsoft Excel 2021. Data were independently extracted using a piloted form, with any discrepancies resolved through consensus. The framework captured general study characteristics (e.g., author, year, study design, population, and sample size) and scoping-specific elements (e.g., theoretical frameworks and key variables).

To facilitate a comparative analysis, participants were categorized into a “direct group” (recipients such as adolescents and adults) and an “indirect group” (parents/guardians). Furthermore, the identified variables were systematically coded into individual and social/structural factors. Individual factors include psychological constructs based on individual beliefs, such as attitude, knowledge, and perceived benefits/barriers, aligning with the core constructs of the TPB and the HBM. In contrast, social/structural factors encompass subjective norms, cues to action, and demographic indicators. Notably, age and sex were categorized as structural factors in this study, as they function as primary indicators of policy eligibility (e.g., NIP targets) and social disparity in the Korean context. The detailed categorization criteria and their theoretical rationales are summarized in Table 1.

### 2.6. Stage 5: Collating, Summarizing, and Reporting the Results

In this final stage, the extracted data were synthesized to map the overall research landscape of HPV vaccination intention in Korea. Descriptive statistics and frequency analysis were employed to summarize the general characteristics of the included studies and the prevalence of specific determinants. Based on the extracted data, we compared the identified variables and trends between the direct (vaccine recipients) and the indirect groups (proxy decision-makers) to identify converging and diverging research patterns. The results are presented through a combination of tabulated summaries and narrative synthesis, highlighting current research trends, methodological gaps, and evidence-based implications for public health policy.

## 3. Results

### 3.1. General Characteristics

An analysis of 36 studies demonstrated that 44.4% of the research on HPV vaccination intention was published between 2016 and 2020, coinciding with the period when the HPV vaccine was incorporated into the NIP in Korea, reflecting increased research interest (Table 2). Studies published after 2020 accounted for 30.6% of the studies, suggesting that interest in HPV vaccines remained steady, even during the coronavirus disease 2019 pandemic. All the included studies adopted quantitative research designs, indicating that HPV vaccination intention Korean research has primarily focused on identifying measurable predictive factors.

When study populations were classified by direct involvement in HPV vaccination, 61.1% of studies targeted the direct group (adults and adolescents who are actual vaccination decision-makers). Conversely, 38.9% focused on the indirect group (parents or guardians), which is expected because parents are key decision-makers for adolescent vaccination; however, this distribution also indicates limited evidence on adolescent perceptions and vaccination intention. Overall, this pattern suggests that Korean HPV vaccination research places greater emphasis on parental decision-making factors than on adolescent autonomous perspectives.

Regarding age distribution, studies involving adults and adolescents in the direct group accounted for 44.4% and 11.1%, respectively. Conversely, in the indirect group, most studies involved parents of elementary (16.7%) and middle school (8.3%) children, and no studies specifically targeted parents of high school students. This distribution suggests a research focus on parental determinants rather than adolescent awareness and HPV vaccination intention.

Regarding sex, among studies in the direct group, those targeting women accounted for 27.8%, those including both men and women accounted for 22.2%, and studies exclusively involving men were extremely scarce. In the indirect group, most studies were conducted with mothers of daughters (22.2%), indicating that research has largely focused on female-related vaccination. These findings highlight a persistent perception of HPV as primarily a cancer affecting women, which likely contributes to the relative lack of research involving men.

Regarding theoretical foundations, 36.1% and 16.7% of the analyzed studies employed the TPB and HBM, respectively, indicating that Korean research on HPV vaccination intention has mainly focused on theory-based predictors derived from established behavioral models, including attitudes, subjective norms, and perceived susceptibility.

### 3.2. Factors Influencing HPV Vaccination Intention

Across the 36 included studies, 13 factors associated with HPV vaccination intention were identified and categorized as individual and social/structural factors (Table 3). Among the individual factors, attitude was the most frequently reported predictor (47.2%), followed by perceived behavioral control (30.6%), knowledge (27.8%), and perceived benefits (22.2%). These variables primarily reflect individuals’ psychological and cognitive characteristics, suggesting that personal perceptions and judgments play a central role in shaping HPV vaccination intentions.

Among social and structural factors, subjective norms demonstrated the highest frequency (38.9%), followed by age (16.7%), sex (11.1%), and socioeconomic status (8.3%). This pattern suggests that, beyond individual characteristics, external influences such as the expectations of significant others and broader sociocultural or environmental conditions jointly contribute to vaccination intention formation.

Overall, the factors related to HPV vaccination intention encompass a complex mix of psychological, social, and demographic factors. Existing studies have primarily focused on these variables to elucidate the underlying structure of intention formation and explain how multiple determinants interact in the decision-making process regarding HPV vaccination.

### 3.3. Comparison of Influencing Factors on HPV Vaccination Intention Between Direct and Indirect Target Groups

Across studies, the distribution of factors influencing HPV vaccination intention varied by study population type (Table 4). In the direct group, consisting of individuals who were the actual recipients of the vaccine, attitude (27.8%) was the most frequently reported predictor, followed by knowledge (16.7%) and subjective norms (13.9%) as key variables. In this group, the cognitive and psychological characteristics of the individuals accounted for a relatively large proportion of the influential factors.

Conversely, in the indirect group, which included parents or guardians who decided whether their children received the vaccine, subjective norms (25.0%) exhibited the highest frequency, with attitude (16.7%) and perceived behavioral control (16.7%) identified as major predictors. Knowledge (5.6%) occurred less frequently than it did in the direct group, and variables related to external influences, such as expectations of significant others and broader social pressures, were relatively more prominent.

Thus, the composition of factors shaping HPV vaccination intention differed between the direct and indirect groups, and the types of variables that exerted a strong influence on each group followed distinct patterns.

## 4. Discussion

In this scoping review, we identified a significant imbalance in Korean HPV vaccination research, characterized by a focus on female and adult populations despite the need for broader coverage.

### 4.1. Population-Based Public Health Research: Expanding Direct Recipient Studies

This analysis revealed a clear imbalance in the study population. Approximately 60% of the included studies focused on adults or adolescents who were direct HPV vaccination recipients (S1, S2, S3, S4, S6, S9, S12, S13, S14, S15, S17, S19, S22, S24, S26, S28, S29, S30, S31, S33, S34, and S36). Contrastingly, the remaining studies were indirect investigations targeting guardians, mainly parents, who made vaccination decisions on behalf of their children (S5, S7, S8, S10, S11, S16, S18, S20, S21, S23, S25, S27, S32, and S35). Notably, in studies on adolescent vaccination, most assessed parents’ vaccination intentions rather than adolescents’ own intentions, highlighting a significant lack of direct research involving adolescents as decision-makers.

This tendency may reflect Korea’s institutional structure, which requires parental consent for vaccination of minors [5], and the ethical sensitivity surrounding HPV vaccination because of its role in preventing sexually transmitted infections [23,24]. It may also stem from limited attention to adolescents’ autonomous awareness and decision-making processes. In contrast, many countries have institutionalized systems in which adolescents above a certain age can independently decide on vaccination or actively participate in information-sharing and consent procedures, resulting in more frequent direct inclusion of adolescents as research participants [25,26,27,28,29]. These studies used diverse methodologies, including qualitative research [25,27,28] and experimental designs [26,29], to examine adolescent perceptions and decision-making regarding HPV vaccination in greater depth.

Accordingly, future research should not rely solely on indirect parent surveys; instead, it should directly include adolescents as participants and adopt diverse methodological approaches to more thoroughly examine how adolescents perceive and decide on HPV vaccination.

### 4.2. Sex-Inclusive Vaccination Policy: Expanding Research on Males

Previous studies showed clear limitations related to sex and age. In the direct group, most studies primarily targeted adult women, whereas studies involving men and male adolescents were relatively scarce. This imbalance appears to reflect a policy context in which HPV vaccination was first introduced in Korea as part of the NIP for females, as well as the common perception of the HPV vaccine as a cervical cancer vaccine [30]. However, according to the World Health Organization [4], HPV infection prevalence and HPV-related cancer burden among males are steadily increasing, highlighting the need to expand vaccination efforts and increase research on male perceptions and attitudes toward HPV vaccination.

This concentration of evidence on women and mothers of daughters limits the applicability of the existing findings to discussions of gender-neutral HPV vaccination policies, particularly given the extremely low vaccination coverage among males in Korea. However, South Korea is currently at a significant juncture, with active legislative and public health discussions underway regarding the expansion of the NIP to include adolescent boys. This shifting policy landscape underscores the urgent need for a robust evidence base to support the successful implementation of gender-neutral vaccination strategies.

Although Korean studies have predominantly focused on females, international research has pursued a more gender-balanced approach. For instance, in countries such as the United States, Australia, and Canada, the HPV vaccine is included in NIPs for both males and females. Consequently, active research has been conducted on vaccination rates, acceptance, and awareness among males and male adolescents in these countries [31,32,33]. Notably, some studies have compared the determinants of vaccination intention according to sex or evaluated the effectiveness of male-specific educational interventions, offering valuable implications for research in Korea [31,34]. These international findings emphasize that the inclusion of males as vaccination recipients and as research participants has become increasingly important in informing sex-inclusive vaccination policies.

Therefore, future studies in Korea should acknowledge the gender disparity surrounding the HPV vaccine and actively expand research involving male and adolescent male populations. Addressing this evidence gap is essential for generating a more balanced knowledge base aiming to support discussions on inclusive vaccination strategies and policy considerations. Notably, such efforts may also enhance vaccine policy inclusiveness and equity.

### 4.3. Beyond Traditional Behavioral Models: Emerging Public Health Determinants

Analysis of the variables presented in Table 3 indicates that determinants of HPV vaccination intention can be broadly categorized as individual and social/structural factors. Attitudes, knowledge, perceived benefits, perceived barriers, perceived susceptibility, perceived severity, self-efficacy, and perceived behavioral control reflect psychological attributes grounded in personal beliefs and cognition. These constructs represent individual-level factors proposed by the TPB and HBM [35,36]. These individual factors are characterized by their association with education and information, and they may be considered in discussions related to educational and counseling approaches targeting adolescents and their parents.

Contrastingly, factors such as subjective norms, cues to action, age, sex, and socioeconomic status are social and structural determinants of HPV vaccination intention that are less subject to individual control and heavily influenced by external environments such as families, peer groups, schools, communities, and the healthcare system [36]. Accordingly, these factors have been discussed within the broader social, institutional, and policy contexts that influence vaccination decisions.

However, most studies reviewed in this scoping review reported selecting variables primarily based on established behavioral theories, such as the TPB and HBM, or they did not explicitly report a theoretical framework, which may have omitted some practical or emerging determinants. As a result, similar theory-driven constructs—such as attitudes, norms, perceived control, and knowledge—were repeatedly examined across studies. Beyond traditional theory-based factors, recent research highlights a growing need to reconsider the role of contextual and structural elements. For example, false or distorted information disseminated via social media platforms such as Twitter and Instagram has been reported to negatively affect HPV vaccination intention and parental vaccine perceptions [37,38], suggesting that the digital information environment should be considered a determinant. Additionally, within Asian contexts, cultural and religious beliefs, gender norms, and traditional values contribute substantially to HPV vaccine hesitancy and refusal. These normative influences are complex and cannot be addressed through knowledge provision alone, underscoring the need for variables that measure and analyze cultural context, such as the perception of HPV as an “issue for women” [39].

By explicitly mapping both the dominance of traditional behavioral models and the limited inclusion of emerging contextual determinants, in this scoping review, we identify critical gaps in the existing evidence base and areas for future investigation.

### 4.4. Consideration of Distinct Patterns of Influencing Factors Between the Direct Group and the Indirect Group

In this study, we identified distinct patterns in the determinants of HPV vaccination intention between the direct and indirect groups. In the direct group, attitude and knowledge emerged as the primary predictors. This suggests that adolescents and adults, as autonomous decision-makers, rely heavily on personal beliefs and information [40,41], reinforcing the importance of individual cognitive mechanisms as outlined in the TPB. Conversely, in the indirect group (parents or guardians), subjective norms and perceived behavioral control were the predominant factors. This shift indicates that proxy decision-making for children is more significantly shaped by perceived social expectations and environmental controllability [42,43,44] rather than by personal cognition alone. These findings highlight that intervention strategies should be tailored specifically to these differing psychological drivers.

These differences between the groups highlight variations in decision-making patterns that may be considered when discussing potential approaches to HPV vaccination promotion. Strategies targeting direct groups may be discussed in relation to information provision and attitudinal factors, as reported in existing studies, without assuming direct effects on vaccination behavior. Specifically, educational interventions that provide accurate, evidence-based information on HPV and related diseases and foster positive attitudes toward vaccination benefits and safety may be effective. Such interventions can be implemented through school-based sexual health education programs and enhanced through peer education to increase adolescents’ engagement and message receptivity.

Conversely, for indirect groups such as parents or guardians, existing studies have primarily emphasized the role of social norms and perceived decision-making contexts in shaping vaccination intention. Approaches that help guardians make responsible decisions, grounded in scientific evidence and the health benefits for their children, are essential. This could include community-based campaigns to promote positive social perceptions of HPV vaccination, strengthening vaccination guidance during school enrollment, and policies that encourage healthcare providers to routinely recommend HPV vaccines during clinical visits.

Overall, our findings highlight the differences in decision-making mechanisms between the direct and indirect groups. These observations indicate that population groups may exhibit distinct patterns of associated factors, warranting further investigation through longitudinal and intervention-based study designs.

#### Strengths and Limitations

This scoping review has some limitations that should be acknowledged. First, as all the studies included in this review were cross-sectional, the findings can only identify associations between variables rather than establish causal relationships. Therefore, the predictors identified in this study should be interpreted as patterns of vaccination intention rather than definitive causes of behavior. Future longitudinal or experimental research is required to clarify these causal links and to evaluate the long-term effectiveness of the identified determinants.

Second, this review was limited to studies indexed in Korean databases. Although this focus was an intentional methodological choice to prioritize a comprehensive mapping of local evidence—including a significant body of gray literature such as academic theses that are often omitted from global databases—it may have excluded Korean studies published in international journals indexed in databases like PubMed or Scopus. Furthermore, the predominance of quantitative research among the included studies limits a deeper understanding of the nuanced ethical and contextual dimensions of vaccination decision-making. Future research should incorporate both global databases and diverse methodologies, such as qualitative and mixed-methods approaches, to provide a more holistic and culturally sensitive perspective on HPV vaccination intention in South Korea.

Despite these limitations, this study has several notable strengths. First, we systematically mapped how traditional behavioral theories—particularly the TPB and the HBM—have been predominantly applied in Korean research on HPV vaccination intention, thereby clarifying patterns of theoretical concentration and construct repetition. Second, by concurrently organizing emerging but underexplored contextual and structural determinants—such as digital misinformation, sociocultural norms, and gender role perceptions—in this review, we delineate the current scope and limitations of the evidence base and identify critical gaps for future investigation.

In addition, we distinguished between direct vaccination decision-makers (direct group) and proxy decision-makers (indirect group), revealing differences in the composition of associated factors across groups. This analytical distinction extends beyond simple aggregation of prior studies and provides a structured perspective on how decision-making mechanisms may vary by population type.

## 5. Conclusions

In this review, we highlight that Korean research on HPV vaccination intention shows systematic patterns in study populations, influencing factors, and decision-making structures, which have direct implications for national vaccination policy and program design. These findings provide an overview of existing research patterns in Korea and may inform future discussions on HPV vaccination policies aimed at supporting the WHO target of 90% vaccination coverage.

Regarding study populations, the predominance of research focused on women or parents underscores the need to expand to include direct studies targeting adolescents, particularly males. This gap reflects a research imbalance and a critical public health issue, considering that the current male vaccination rate remains as low as 0.2% and that no gender-neutral immunization policy is available in place. Because sex-neutral vaccination has been reported in the international literature as being associated with improved herd immunity and with cost-effectiveness, the expansion of HPV vaccination policies to include males has been increasingly discussed and may warrant careful consideration in future policy deliberations in Korea.

Additionally, we identified individual factors (perceived benefits, perceived susceptibility, and self-efficacy) and social/structural factors (subjective norms and cues to action) as important variables influencing HPV vaccination intention. The analysis also showed that influencing factors differed between the “direct group” and the “indirect group”, providing a basis for tailored interventions aligned with group-specific characteristics. Moreover, international studies have identified emerging variables—such as misinformation on social media, gender role norms, and cultural beliefs—that are not fully captured by traditional theoretical models, including the HBM and TPB. Future research should actively examine these contextual and latent variables.

## Figures and Tables

**Table 1:** Categorization of Variables Influencing HPV Vaccination Intention.

Category	Definition	Included Variables	Theoretical Rationale
Individual Factors	Psychological characteristics based on individual beliefs and perceptions.	Attitude, Knowledge Perceived benefits Perceived barriers, Perceived susceptibility Perceived severity Self-efficacy, Perceived behavioral control	Aligned with core constructs of TPB and HBM.
Social/Structural Factors	Factors reflecting social expectations, environment, and policy-related constraints.	Subjective norms Cues to action Age Sex Socioeconomic status	Reflects social norms and NIP policy eligibility in Korea.

HPV = Human papillomavirus; TPB = Theory of Planned Behavior; HBM = Health Belief Model; NIP = National Immunization Program.

**Table 2:** General characteristics of included studies (N = 36).

Variables	Categories		N (%)
Published year	>2015		9 (25.0)
	2016~2020		16 (44.4)
	2020~<		11 (30.6)
Study design	Quantitative		36 (100)
	Qualitative		0 (0)
Participant group	Direct target group		22 (61.1)
	Indirect target group		14 (38.9)
Target population (years)	Direct (n =22)	Adolescents (<19)	4 (11.1)
		Adult (≥19)	16 (44.4)
		Adolescents + Adult	2 (5.6)
	Indirect (n = 14)	Elementary school (7~12)	6 (16.7)
		Middle school (13~15)	3 (8.3)
		High school (16~18)	0 (0)
		Elementary to high school (7~18)	5 (13.9)
Sex	Direct (n = 22)	Male	4 (11.1)
		Female	10 (27.8)
		Male + Female	8 (22.2)
	Indirect (n = 14)	Male (son)	2 (5.6)
		Female (daughter)	8 (22.2)
		Male + Female	4 (11.1)
Theory	Theory of Planned Behavior		13 (36.1)
	Health Belief Model		6 (16.7)
	None		17 (47.2)

Direct group: in individuals directly targeted for HPV vaccination. Indirect group: parents or guardians making decisions on behalf of others.

**Table 3:** Frequency of Significant Predictors of HPV Vaccination Intention (N = 36).

Category	Factor	n (%)	Study Number
Individual factor	Attitude	17 (47.2)	1, 7, 8, 14, 15, 16, 17, 18, 19, 20, 23, 26, 27, 29, 30, 34, 36
	Perceived behavior control	11 (30.9)	6, 7, 8, 16, 17, 18, 20, 23, 30, 34, 36
	Knowledge	10 (27.8)	2, 4, 9, 10, 11, 13, 24, 27, 28, 31
	Perceived benefits	8 (22.2)	1, 5, 13, 15, 21, 28, 33, 35
	Self-efficacy	7 (19.4)	1, 19, 23, 25, 27, 28, 32
	Perceived barriers	6 (16.7)	5, 15, 21, 28, 31, 35
	Perceived susceptibility	4 (11.1)	10, 14, 33, 35
	Perceived severity	4 (11.1)	1, 13, 33, 35
Socio-Structural factor	Subjective norm	14 (38.9)	6, 7, 8, 16, 17, 18, 19, 20, 23, 25, 27, 30, 32, 36
	Age	6 (16.7)	1, 2, 3, 4, 8, 19
	Sex	4 (11.1)	19, 24, 33, 36
	Socioeconomic state	3 (8.3)	1, 4, 10
	Cue to action	1 (2.8)	28

HPV: Human papillomavirus. Note. N (%) indicates the number and percentage of included studies in which each factor was identified as a significant predictor of HPV vaccination intention. Percentages are based on the total number of included studies (N = 36).

**Table 4:** Comparison of Significant Predictors of HPV Vaccination Intention Between Direct and Indirect Target Groups (N = 36).

Direct Group			Indirect Group		
Factor	N (%)	Study Number	Factor	N (%)	Study Number
Attitude	10 (27.8)	1, 14, 15, 17, 19, 26, 29, 30, 34, 36	Subjective norm	9 (25.0)	7, 8, 16, 18, 20, 23, 25, 27, 32
Knowledge	6 (16.7)	2, 4, 9, 13, 24, 31	Attitude	6 (16.7)	7, 8, 16, 18, 20, 23
Subjective norm	5 (13.9)	6, 17, 19, 30, 36	Perceived behavior control	6 (16.7)	7, 8, 16, 18, 20, 23
Perceived behavior control	5 (13.9)	6, 17, 30, 34, 36	Perceived benefits	3 (8.3)	5, 21, 35
Perceived benefits	5 (13.9)	1, 13, 15, 28, 33	Perceived barriers	3 (8.3)	5, 21, 35
Perceived susceptibility	3 (8.3)	14, 28, 33	Self-efficacy	3 (8.3)	23, 25, 32
Perceived severity	3 (8.3)	1, 13, 33	Perceived susceptibility	2 (5.6)	10, 35
Perceived barriers	3 (8.3)	15, 28, 31	Knowledge	2 (5.6)	10, 11
Self-efficacy	3 (8.3)	1, 19, 28	Perceived severity	1 (2.8)	35
Cue to action	1 (2.8)	28	Cue to action	0 (0.0)	

Direct group: in individuals directly targeted for HPV vaccination. Indirect group: parents or guardians making decisions on behalf of others. N (%): Number and percentage of studies in which the factor was identified as a significant predictor, calculated based on the total number of included studies (N = 36).

## Data Availability

No new data were created or analyzed in this study. Data sharing is not applicable to this article.

## Supplementary Materials

The following supporting information can be downloaded at: https://www.mdpi.com/article/10.3390/healthcare14030355/s1. File S1: Articles selected in this study for scoping review. File S2: Data extraction from the included studies. File S3: JBI Critical Appraisal Checklist. Refs. [45,46,47,48,49,50,51,52,53,54,55,56,57,58,59,60,61,62,63,64,65,66,67,68,69,70,71,72,73,74,75,76,77,78,79] are cited in the Supplementary Materials.

## Abbreviations

HBM	Health Belief Model
HPV	Human papillomavirus
JBI	Joanna Briggs Institute
KISS	Korean Studies Information Service System
NDSL	National Digital Science Library
NIP	National Immunization Program
PCC	Population, Concept, Context
RISS	Research Information Sharing Service
TPB	Theory of Planned Behavior
WHO	World Health Organization
