# Supplementary material for: Research Trends and Gaps in Human Papillomavirus Vaccination Intention in South Korea: A Scoping Review
Source: Healthcare (Basel). 2026 Jan 30;14(3):355. doi: 10.3390/healthcare14030355 (PMC12896974; doi:10.3390/healthcare14030355)
Supplement: Supplementary file 1 [file healthcare-14-00355-s001.zip › File S1.pdf]

Supplementary S1. Articles selected in this study for scoping review

S1. Cho, S. Predicting women`s cervical cancer prevention behaviors extending health belief model. *J Korean Soc Advert Educ*. **2011**, *91*, 348-377.

S2. Kim, H.W. Comparison of factors associated with intention to receive human papillomavirus vaccine between male and female undergraduate students. *Korean Journal of Women Health Nursing*. **2011**, *17*(4), 415-425. DOI:10.4069/kjwhn.2011.17.4.415

S3. Lee, J. Human papillomavirus vaccination acceptance and factors influencing in female university students [master's thesis]. Chung-Ang University; 2011.

S4. Kang, S.W.; Jun, E.M. The study of human papilloma virus vaccination acceptance in marrid women, *Journal of the Korean Data Anaysis Society*. **2013**, 15(1), 237-249.

S5. Lee, Y.L. Factors influencing intention for human papillomavirus vaccination among mothers with female adolescent [master's thesis]. Ajou Unitersith; 2014

S6. Lee, K.E. Factors associated with intention to receive human papillomavirus vaccine in undergraduate women: An aplication of the theory of planned behavior *Journal of Korean Academy of Fundamentals of Nursing*. **2014**, 21(4), 457-465. DOI:10.7739/jkafn.2014.21.4.457

S7. Park, H.M.; Oh, H.E. Factors associated with the intention of human papillomavirus vaccination among mothers of junior high school daughters. *The Journal of the Korea Contents Association*. **2014**, 14(8), 307-318. DOI:10.5392/JKCA.2014.14.08.307

S8. Lee, J.H. Factors associated with the intention of human papillomavirus vaccination among parents of the middle school children, [master's thesis]. Chung-Ang University; **2015**.

S9. Lee, S.Y.; Han, M.A.; Park, J.; Ryu, S.Y. Factors associated with human papillomavirus vaccination and intention among male and female college students. *Korean journal of health promotion*. **2015**, 15(3), 141-149. DOI:10.15384/kjhp.2015.15.3.141

S10. Shim, J.L.; Ha, Y.J. Factors influencing intention for human papillomavirus vaccination among parents with

elementary school girls. *The Journal of Korean Academic Society of Nursing Education*. **2017**, 23(4), 367-377. DOI:10.5977/jkasne.2017.23.4.367

S11. Park, S.; Jang, I. Factors influencing practice and intention of HPV vaccination among adolescent daughter's mothers: Focusing on HPV knowledge and sex-related communication. *Journal of the Korean Society of School Health*. **2017**, 30(2), 93-102. DOI:10.15434/kssh.2017.30.2.93

S12. Cho, S. A Sstudy on the relationship between cervical cancer preventive behavioral intentions and optimistic bias among korean female college students. *Korean Journal of Child Studies*. **2018**, 26(3), 101-124. DOI:10.23875/kca.26.3.5

S13. Jung, S.W. A convergence study of cervical cancer knowledge and health belief affecting male non-health related majoring students' HPV vaccination intentions. *Journal of the Korea Convergence Society*. **2018**, 9(3), 289-295. DOI:10.15207/JKCS.2018.9.3.289

S14. Jang, I. Comparison of factors associated with intention to HPV vaccination between male and female high school students: Focusing on HPV knowledge, attitude and health beliefs related to HPV. *Journal of the Korean Society of School Health*. **2018**, 31(2), 59-69. DOI: 10.15434/kssh.2018.31.2.59

S15. Yun, Y.; Koh, C.K. Factors influencing human Pppillomavirus vaccination intention among unvaccinated nursing students in korea. *The Journal of Korean Academic Society of Nursing Education*. **2018**, 24(3), 205-213. DOI:10.5977/jkasne.2018.24.3.205

S16. Oh, Y.J.; Lee, E.M. Convergence related factors and HPV vaccination intention for mothers with children elementary school. *Journal of digital convergence*. **2018**, 16(3), 311-319. DOI:0.14400/JDC.2018.16.3.311

S17. Sung, M.H.; Sung, M.H. Factors influencing human papillomavirus vaccination intention in female high school students: Application of planned behavior theory. *Korean journal of women health nursing*. **2018**, 24(1), 71-79. DOI: 10.4069/kjwhn.2018.24.1.71

S18. Park, H.S. Factors associated with parental inention to vaccinate adolescent daughters against HPV [master's thesis]. Seoul National University; **2018**.

S19. Kim, S.Y. Factors influencing the human papilloma virus vaccination using the theory of planned behavior

in college students, [master's thesis]. Chung-Ang University: **2018**.

S20. Hong, S.H. Factors affecting the intentions and behavior of human papilloma virus vaccination in adolescent daughters. *Journal of the Korea Contents Association*. **2019**, 19(1), 223-233. DOI:10.5392/JKCA.2019.19.01.223

S21. Hong, S.H.; Chung, Y.H. Predictors of human papillomavirus vaccination of female adolescent mothers. *Journal of digital convergence*. **2019**, 17(4), 149-157. DOI:10.14400/JDC.2019.17.4.149

S22. Joo, W. A study on the factors affecting human papillomavirus vaccination [master's thesis]. University of Ulsan; **2019**.

S23. Kang, E.H. Factors associated with parental intention to vaccinate elementary school boys against HPV: Based on the theory of planned behavior [master's thesis]. Ajou University; **2019**.

S24. Han, J.Y.; Kim, S.Y.; Lee, C.M.; Jeong, C.R.; Kim, S.; Sung, K.W. Factors affecting sexual knowledge and attitude on intentions to receive human papilloma virus (HPV) vaccination in male and female university students. *Global Health and Nursing*. **2020**, 10(1), 69-78. DOI: 10.35144/ghn.2020.10.1.69

S25. Park, E.Y.; Kim, T.I. Factors influencing mothers' intention to vaccinate their elementary school sons against human papillomavirus. *Korean Journal of Women Health Nursing*. **2020**, 26(1), 37-48. DOI:10.4069/kjwhn.2020.03.07

S26. Lee, Y.H.; Park, K.O. Factor associated with the intention of human papillomavirus vaccination with nursing students: Based on the theory of planned behavior. *Journal of the Korea Convergence Society*. **2021**, 12(9), 343-350. DOI: 10.15207/JKCS.2021.12.9.343

S27. Nam, K.A.; Lee, Y.E. Influencing factors on intention to receive human papillomavirus vaccination in mothers of elementary school girls: Focusing on the mediating effects of self-efficacy. *Journal of Korean Society of Maternal and Child Health*. **2021**, 25(2), 130-141. DOI:/10.21896/jksmch.2021.25.2.130

S28. Kim, S.I. A structural equation model of male high school students human papilloma virus vaccination [master's thesis]. Korea University; **2024**.

S29. Son, Y.; Ahn, O. Factors associated with intention to receive HPV vaccination among marriage-immigrant

women. *Journal of the Korea Academia-Industrial cooperation Society*. **2021**, 22(5), 465-474. DOI:10.5762/KAIS.2021.22.5.465

S30. Jang, S.H. Factors influencing the Human Papilloma Virus vaccination(HPV) intention of male fresh student in university. *Journal of Learner-Centered Curriculum and Instruction.* **2022**, 22(15), 331-342. DOI:10.22251/jlcci.2022.22.15.331

S31. Lee, M.;Kim, S. Factors affecting HPV(Human Papilloma Virus) vaccination intentions of female adolescents and their mothers. *Journal of Korean Public Health Nursing*. **2022**, 36(1), 33-46. DOI:10.5932/JKPHN.2021.36.1.33

S32. Lee, J.Y. Factors affecting HPV vaccination to the children of married immigrant women [Doctoral dissertation]. Yonsei University; **2021**.

S33. Hong, D.Y.; Kim, S.Y.; Kim, Y.E.; Seok, M.K. Lim, K.H. Factors affecting intention to HPV vaccination of a university students. *Nursing and Healthvare Science.* **2023**, 22(2), 83-89. DOI:10.12972/nhs.20230010

S34. Cao, Y. Factors affecting the intention to vaccinate human papillomavirus (HPV) by applying the Theory of Planned Behavior- for adult male [master's thesis]. Kyung Hee University; **2023**.

S35. Chang, J.H. The effect of article message types on parents' intentions for Human Papillomavirus(HPV) vaccination for elementary school children: Focusing on the health belief model [master's thesis]. Sogang University; **2023**.

S36. Kim, K.O. Factors influencing high school students' human papilloma virus vaccination intention: Based on the theory of planned behavior [master's thesis]. Chonnam National University; **2024**.
