# Supplementary material for: Research Trends and Gaps in Human Papillomavirus Vaccination Intention in South Korea: A Scoping Review
Source: Healthcare (Basel). 2026 Jan 30;14(3):355. doi: 10.3390/healthcare14030355 (PMC12896974; doi:10.3390/healthcare14030355)
Supplement: Supplementary file 1 [file healthcare-14-00355-s001.zip › File S2.pdf]

Supplementary S2. Data extraction from the included studies

No	Author (yr)	Age		Gender		Sample (n)	Theory	Result
		Direct	Indirect	Direct	Indirect			
S1	Cho S (2011)	18~30		F		1190	HBM	Age, Socioecomonic state , Attitude, Perceived benefits, Self-efficacy, Perceived severity
S2	Kim HW (2011)	20.7		Both		479		Age, Knowledge, Knowledge
S3	Lee J (2012)	19.9		F		189		Age
S4	Kang SW, Jun EM (2013)	24~61		F		102		Age, Socioecomonic state, Knowledge, Knowledge
S5	Lee YL (2014)	44.2	10~18	F	F	205		Perceived benefits, Perceived barriers
S6	Lee KE (2014)	20<		F		254	TPB	Subjective norm, Perceived behavior control
S7	Park HM, Oh HE (2014)	44.3	14~15	F	F	198	TPB	Attitude, Subjective norm, Perceived behavior control
S8	Lee JH (2015)	30<	14~15	Both	Both		TPB	Age, Attitude, Subjective norm, Perceived behavior control
S9	Lee SY, Han MA, Park J, Ryu SY (2015)	20.4		Both		489		Knowledge
S10	Shim JL, Ha YJ	20~50	9~12	Both	F	210		Socioecomonic state, Knowledge, Perceived

	(2017)							susceptibility
S11	Park S, Jang I (2017)	45.8	12~19	F	F	298		Knowledge
S12	Cho S (2018)	18~30		F		1191		
S13	Jung SW (2018)	21		M		159		Knowledge, Perceived benefits, Perceived severity
S14	Jang I (2018)	17		Both				Attitude, Perceived susceptibility
S15	Yun Y, Koh CK (2018)	20<		F		205		Attitude, Perceived benefits, Perceived barriers
S16	Oh YJ, Lee EM (2018)	39.6	7~12	F	F	132	TPB	Attitude, Subjective norm, Perceived behavior control
S17	Sung MH, Sung MH (2018)	16~17		F		138	TPB	Attitude, Subjective norm, Perceived behavior control
S18	Park HS (2018)	43.3	12~14	Both	F	138	TPB	Attitude, Subjective norm, Perceived behavior control
S19	Kim SY (2018)	18~26		Both		778	TPB	Age, Gender, Attitude, Subjective norm, Self-efficacy
S20	Hong SH (2019)	20<	13~14	F	F	249	TPB	Attitude, Subjective norm, Perceived behavior control
S21	Hong SH, Chung YH (2019)	40~50	12~13	F	F	285	HBM	Perceived benefits, Perceived barriers
S22	Joo W (2019)	10~50		F		183		

S23	Kang EH (2019)	30~49	11~12	Both	M	231	TPB	Attitude, Subjective norm, Perceived behavior control, Self-efficacy
S24	Han JY, Kim SY, Lee CM, Jeong CR, Kim S, Sung KW (2020)	23<		Both		179		Gender, Knowledge
S25	Park EY, Kim TI (2020)	42.7	10~12	F	M	151		Subjective norm, Self-efficacy
S26	Lee YH, Park KO (2021)	22.1		Both		195	TPB	Attitude
S27	Nam KA, Lee YE (2021)	42.4	11~12	F	F	171		Attitude, Subjective norm, Knowledge, Self-efficacy
S28	Kim SI (2021)	15~18		M		330	HBM	Knowledge, Perceived benefits, Self-efficacy, Perceived barriers, Cue to action
S29	Son Y, Ahn O (2021)	20~40		F		136		Attitude
S30	Jang SH (2022)	18<		M		162	TPB	Attitude, Subjective norm, Perceived behavior control
S31	Lee M, Kim S (2022)	20<	12	F	F	64	HBM	Knowledge, Perceived barriers
S32	Lee JY (2022)		9~18	F	Both	262	HBM	Subjective norm, Self-efficacy
S33	Hong DY, Kim SY, Kim YE, Seok MK, Lim KH (2023)	20<		Both		122		Gender, Perceived benefits, Perceived susceptibility Perceived severity,

S34	CAO Y (2023)	20~39		M		500	TPB	Attitude, Perceived behavior control,
S35	Chang JH (2024)	30~50	7~12	Both	Both	200	HBM	Perceived benefits, Perceived barriers, Perceived susceptibility, Perceived severity
S36	Kim KO (2024)	18		Both		192	TPB	Gender, Attitude, Subjective norm, Perceived behavior control,
